# Constraints from the dehydration of antigorite on high-conductivity anomalies in subduction zones

**DOI:** 10.1038/s41598-017-16883-4

**Published:** 2017-12-04

**Authors:** Duojun Wang, Xiaowei Liu, Tao Liu, Kewei Shen, David O. Welch, Baosheng Li

**Affiliations:** 1 College of Earth Sciences, University of Chinese Academy of Sciences, Beijing, 100049 China; 2 Mineral physics institute, State university of New York at Stony Brook, Stony Brook, 11794 NY, USA; 3 Laboratory of Mechanics on Disaster and Environment in Western China, Lanzhou University, Lanzhou, 730000 China; 4 Condensed Matter Physics and Materials Science Department, Brookhaven National Laboratory, Upton, 11793 USA

## Abstract

Regions with high electrical conductivities in subduction zones have attracted a great deal of attention. Determining the exact origin of these anomalies could provide critical information about the water storage and cycling processes during subduction. Antigorite is the most important hydrous mineral within deep subduction zones. The dehydration of antigorite is believed to cause high-conductivity anomalies. To date, the effects of dehydration on the electrical conductivity of antigorite remain poorly understood. Here, we report new measurements of the electrical conductivity of both natural and hot-pressed antigorite at pressures of 4 and 3 GPa, respectively, and at temperatures reaching 1073 K. We observed significantly enhanced conductivities when the antigorite was heated to temperatures beyond its thermodynamic stability field. Sharp increases in the electrical conductivity occurred at approximately 848 and 898 K following the decomposition of antigorite to forsterite, enstatite and aqueous fluids. High electrical conductivities reaching 1 S/m can be explained by the presence of an interconnected network of conductive aqueous fluids. Based on these results for the electrical conductivity of antigorite, we conclude that high-conductivity regions associated with subduction zones can be attributed to dehydration-induced fluids and the formation of interconnected networks of aqueous fluids during the dehydration of antigorite.

## Introduction

**Figure 1:**
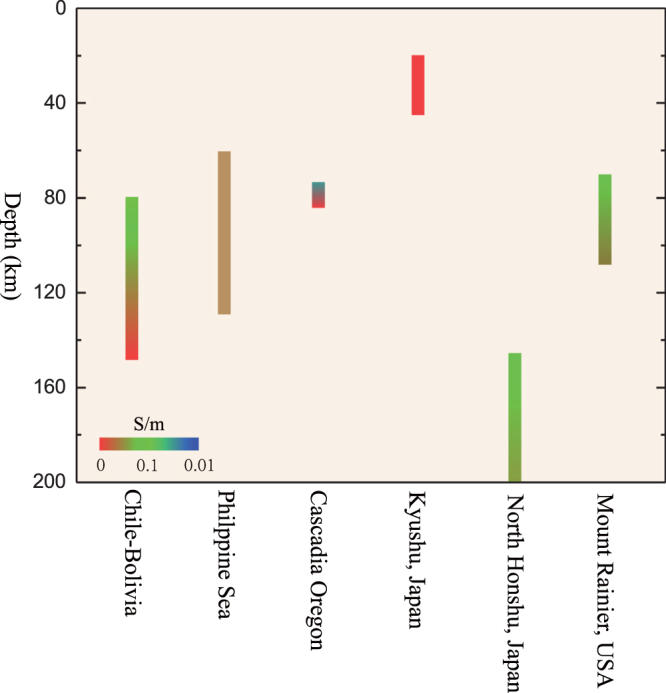
Typical high electrical conductivity (0.1~1 S/m) regions associated with subduction zones, These regions include the Chile-Bolivia region^5^, the Philippine Sea^6^, the Cascadia Oregon^7^, the areas of Kyushu, Japan^8^ and North Honshu, Japan^9^, and Mount Rainier, USA^10^.

The water liberated from the breakdown of hydrous minerals within a subduction zone may be transported to the overlying mantle wedge and subsequently trigger magmatism^1,2^, thereby inducing seismic^3,4^ and magnetotelluric (MT) anomalies^5–15^ above the subduction zone. Many electromagnetic studies^5–15^ have revealed anomalously high electrical conductivities (0.1–1 S/m) in backarc regions as well as in forearc regions in subduction settings (Fig. 1). These anomalies (e.g., extremely high-conductivity values of ~1 S/m) cannot be entirely ascribed to the presence of hydrated olivine and pyroxene^16–20^ but have often been attributed to the presence of the aqueous fluids released by hydrous minerals in the subducting slabs.

Serpentine minerals are the most abundant hydrous minerals in altered ultramafic rocks^21^. Serpentine minerals are also believed to compose over 90% of the hydrated upper mantle^22^. Among them, antigorite is the only stable serpentine mineral at depths reaching 200 km in deep subduction zones. Antigorite, Mg_3_Si_2_O_5_(OH)_4_, contains up to 15–16% (average 13 wt %) water as structural hydroxyl (OH^−^) groups. The presence of serpentine and other hydrous minerals affects the physical and mechanical properties of a peridotite mantle, such as the seismic velocity, density, and mechanical strength^21,23–25^.

However, the electrical conductivities of antigorite and other hydrous minerals are relatively low (~10^−4^ S/m) at temperatures below 773 K^26–28^. These low values cannot effectively explain geophysically observed anomalies consisting of high electrical conductivity values. The dehydration of certain hydrous minerals, including talc, amphibole, lawsonite and chlorite, can significantly enhance the electrical conductivity anomaly^29–32^. However, although it constitutes the only stable hydrous phase down to a depth of 200 km, the electrical conductivity of dehydrated antigorite has not yet been investigated. Given that a large amount of water is released during this process, dehydration is expected to impose significant effects on the physical properties of antigorite. Therefore, in this paper, we report the electrical conductivity of antigorite both before and after dehydration and apply the results to an explanation of the high electrical conductivity anomalies observed during geophysical surveys in subduction settings.

## Results

**Figure 2:**
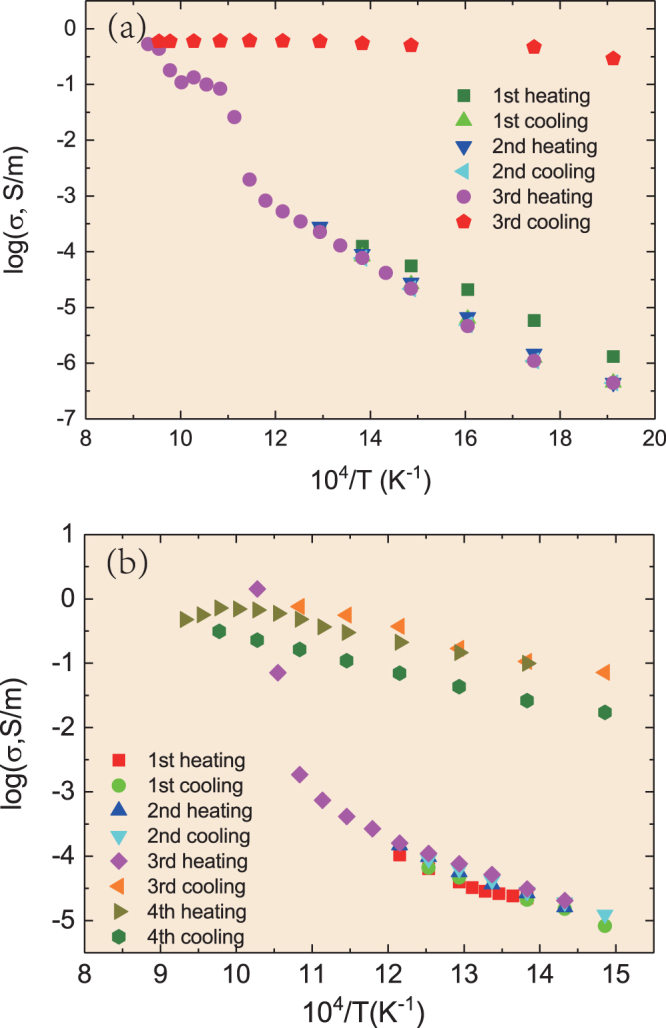
Electrical conductivity of antigorite before, during, and after dehydration. (**a**) Logarithm of the electrical conductivity as a function of the reciprocal of the temperature for natural antigorite during the three heating and cooling cycles at 4 GPa. The symbols represent the data points for the different heating and cooling cycles. (**b**) Logarithm of the electrical conductivity as a function of the reciprocal of the temperature for hot-pressed antigorite during the three heating and cooling cycles at 3 GPa. The symbols represent the data points for the different heating and cooling cycles.

The electrical conductivities of both natural and hot-pressed antigorite were measured at 4 GPa and 3 GPa, respectively, from 523 K to 1073 K (Fig. 2). The results indicate that the electrical conductivity of antigorite has a weak or non-existent pressure dependence but varies significantly with temperature. Three distinct stages of variations in the electrical conductivity were observed, including prior to dehydration, during dehydration and after dehydration. In the first stage (prior to dehydration), the electrical conductivity of antigorite increased steadily with the temperature up to 848 K and 898 K for the natural and hot-pressed samples, respectively. At temperatures of 873–973 K, the electrical conductivity of the natural sample abruptly increased by more than two orders of magnitude to ~0.1 S/m, which was followed by a conductivity increase of approximately one order of magnitude (up to 1 S/m) when the temperature was increased from 973 to 1073 K. Similarly, sharp increases in the electrical conductivity of the hot-pressed sample occurred at temperatures exceeding 923 K, resulting in an increase of three orders of magnitude to a value of 1 S/m at 973 K. The above mentioned temperatures at which increases in the electrical conductivities were observed correspond to the dehydration temperatures of antigorite^22^.

## Discussion

Dehydration releases aqueous fluids, and therefore, the electrical conductivity of a region will be anomalously high if (i) the released aqueous fluids have high conductivities and (ii) these fluids are interconnected. In our experiments, the electrical conductivity values measured during the final cooling stage subsequent to dehydration were significantly higher than those during the corresponding heating path, and the temperature dependence of conductivity was low during this stage. The sharp increase in the electrical conductivity at approximately 873–1073 K coincides with the dehydration of antigorite to the minerals forsterite and enstatite and aqueous fluids. Therefore, we conclude that the observed high-conductivity anomaly subsequent to dehydration was caused by the presence of interconnected regions of fluids.

**Figure 3:**
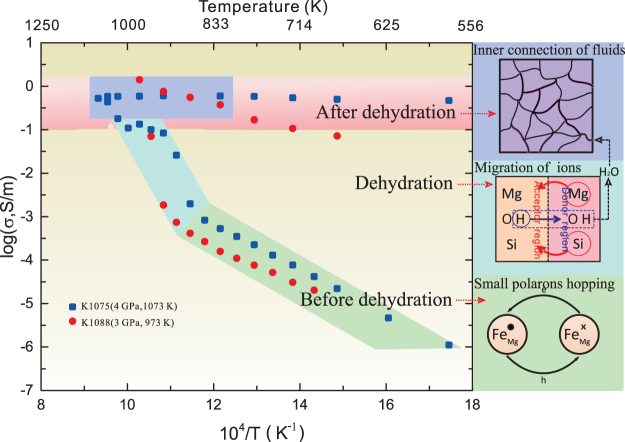
A comparison of the electrical conductivity results (left) of natural and hot-pressured antigorite at 4 and 3 GPa, respectively, with the conduction mechanisms (right) for the different stages. Squares represent the electrical conductivity of natural antigorite at 4 GPa during the third cycle. Circles represent the electrical conductivity of hot-pressed antigorite at 3 GPa during the third cycle. The three conduction mechanisms corresponding to the three stages (prior to dehydration, during dehydration, and after dehydration) are shown in the panel to the right figure. **Bottom panel:** In lower-temperature regions (prior to dehydration), the conduction mechanism is small polaron hopping, i.e., $F{e}_{Mg}^{x}+{h}^{\ast }\leftrightarrow F{e}_{Mg}^{\ast }$. **Middle panel:** The dehydration of antigorite occurs during this stage. Water is formed through a heterogeneous mechanism via combinations between protons in acceptor regions and OH- ions in donor regions. To maintain electrical neutrality, Mg and Si ions in the donor regions move towards acceptor regions. The electrical conductivity is governed by the migration of Mg and Si ions that are associated with large activation energies. **Top panel:** Dehydration-induced aqueous fluids accumulate and form interconnected networks in high-temperature regions. The conductivity is dominated by interconnected networks of conductive aqueous fluids independently of the temperature.

Three distinct trends in the activation enthalpy were observed (Table 1). Prior to dehydration, the activation enthalpy was ~90 kJ/mol, which primarily corresponds to the migration of electron holes between Fe^2+^ and Fe^3+^ ions (as shown in the bottom panel of Fig. 3). During the dehydration process, the observed activation energy was very high (up to 503–991 kJ/mol), which is similar to the values (400–800 kJ/mol) obtained during dehydration kinetic experiments at pressures in the range 100–300 MPa^33^. The dehydration reaction of antigorite is most likely dominated by heterogeneous mechanisms, where the processes involving the migration of ions can be summarized as

1 $${H}_{acceptor\,regions}^{+}+O{H}_{donor\,regions}^{-}\to {H}_{2}{O}_{donorregions}$$

2 $$M{g}_{donor\,regions}^{2+}\to M{g}_{acceptor\,regions}^{2+}$$

3 $$S{i}_{donor\,regions}^{4+}\to S{i}_{acceptor\,regions}^{4+}$$

In these heterogeneous mechanisms (illustrated in the middle panel of Fig. 3), protons in the acceptor regions move outwards and combine with OH^−^ ions in donor regions to form H_2_O molecules; meanwhile, Mg and Si ions in the donor regions undergo counter migration towards acceptor regions to maintain electrical neutrality^34,35^. Migration of Mg and Si ions should contribute to electrical conductivity during this process. The consistency between the activation energies determined herein with those from previous studies^33^ indicates that the conduction mechanism may be a governing factor in the kinetics of the dehydration process. Furthermore, the high activation energy values indicate that the electrical conductivity is controlled by cations (such as Mg and Si ions) rather than protons.

**Table 1:** Results for the activation enthalpy ΔH and the pre-exponential factors for hydrous antigorite and the aqueous fluids.

Runs	Temperature (K)	logA (S/m)	A	ΔH (kJ/mol)	Remarks
K1075	Before dehydration				
	523–773	1.24(0.08)	17	71(1)	1^st^ heating
	523–848	2.24(0.16)	174	88(2)	
	During dehydration				
	873–923	27.56(5.85)	3.6E + 27	503(99)	
	948–1073	5.40(1.38)	2.5E + 5	119(27)	
	After dehydration				
	1048–498	0.064(68)	1.2	5(1)	
K1088	Before dehydration				
	673–898	1.65(0.30)	45	86(4)	
	During dehydration				
	923–973	53.51(2.25)	3.2E + 53	991(40)	
	After dehydration				
	673–923	2.81(0.24)	645	52(4)	3^rd^ cooling
	723–1023	2.15(0.11)	141	44(2)	4^th^ heating
	673–1023	1.96(0.08)	91	48(2)	4^th^ cooling

The activation enthalpy for conduction subsequent to dehydration was very small (5–40 kJ/mol), likely because the electrical conductivity is controlled by the motion of conductive ions in the aqueous fluids (shown in the top panel of Fig. 3).

**Figure 4:**
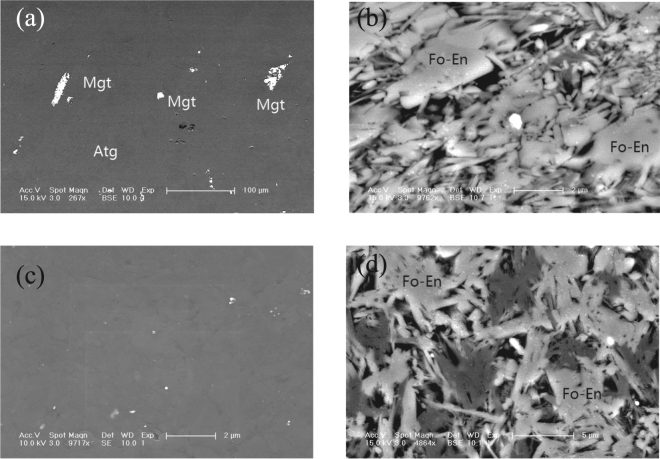
Back-scattered electron (BSE) images of antigorite before and after the electrical conductivity measurements were collected. (**a**) Natural antigorite before electrical conductivity measurements; (**b**) natural antigorite after electrical conductivity measurements; (**c**) hot-pressed antigorite before electrical conductivity measurements; (**d**) hot-pressed antigorite after electrical conductivity measurements. Atg: antigorite, Mgt: magnetite, Fo: Forsterite, En: Enstatite.

New phases, including forsterite and enstatite, were formed as a result of the dehydration, which was confirmed through back-scattered electron (BSE) images (Fig. 4). Fourier-transform infrared (FTIR) spectroscopy measurements (Fig. S1) showed that the absorption peaks of the OH dipoles decreased significantly after electrical conductivity measurements were acquired at 1073 K. Additionally, a very broad absorption peak was observed, suggesting that the released water was partially trapped within the specimens, thereby facilitating the development of an interconnected network along the grain boundaries. Aqueous fluids derived from antigorite are rich in fluid-mobile elements (FME, e.g., B, Li, Cl, As, Sb, Ba, and T)^36–38^ and are highly conductive, leading to a significant enhancement in their electrical conductivity. Therefore, we infer that aqueous fluids rich in conductive ions caused the sharp increase in the electrical conductivity above 873 K. The geometry of fluid/melt (dihedral angle) at the grain boundary plays an important role in forming the connected network. The aqueous fluid forms a connected network affecting the bulk conductivity if the dihedral angle is less than 60°^31,39,40^. In this study, the dihedral angle with the median angle of 52° was observed, suggesting that the aqueous fluids are inter-connected. Given the nature composition of the current samples, the 52° wetting angle is somewhat consistent with the finding of 42° wetting angle on quartzite with saline H_2_O^39^.

Once the aqueous fluids developed an interconnected network, the electrical conductivity exhibited a weak temperature dependency.

Many differences are noted among the electrical conductivities of the dehydration-induced aqueous fluids derived from hydrous minerals. The aqueous fluids released from certain hydrous minerals, including talc^29^, amphibole^30^, epidote^41^, and chlorite^32^, are less conductive. In contrast, the aqueous fluids released from lawsonite^31^ and phlogopite^42^ are highly conductive. These observations reveal that only conductive dehydration-induced aqueous fluids play an important role in modifying the bulk electrical conductivity. Antigorite and chlorite contain up to 13 wt% water, and therefore, interconnected networks of aqueous fluids will form much more easily than within other hydrous minerals. The results from both our study and a previous study^31^ indicate that antigorite and chlorite, which are typically characterized by high, temperature-independent electrical conductivities, can both form interconnected networks of aqueous fluids. However, such properties have not been observed for other hydrous minerals. Therefore, the observed field anomaly at great depth, which was likely sourced from an interconnected network of aqueous fluids, could not have originated from the dehydration of other hydrous minerals.

**Figure 5:**
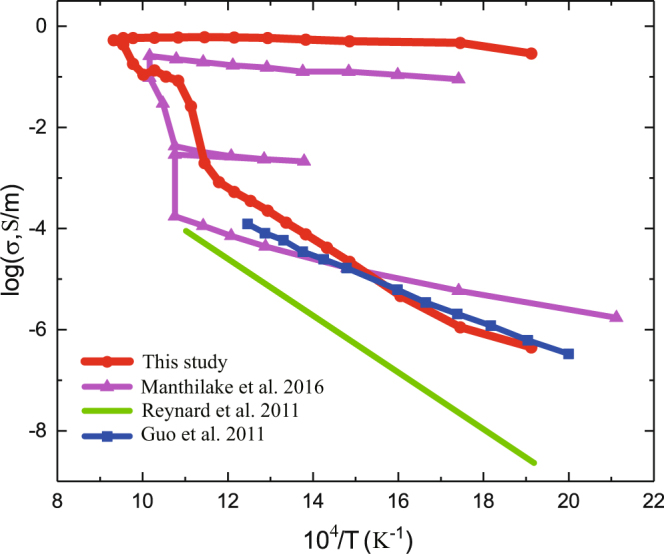
A comparison of the electrical conductivities of hydrous minerals. Circles denote the electrical conductivity of antigorite from this study. Solid line is the electrical conductivity of antigorite from Renard *et al*.^26^. Squares are the electrical conductivities of deformed antigorite from Guo *et al*.^27^. Triangles are the electrical conductivities of chlorite from Manthilake *et al*.^32^. The sharp increases are due to aqueous fluids, and the subsequent enhancements are caused by interconnected magnetite inclusions.

Figure 5 shows a comparison of the electrical conductivities of typical hydrous minerals, including antigorite and chlorite. To date, direct measurements of the electrical conductivity of antigorite have been limited, and only the electrical conductivity of antigorite prior to dehydration has been measured. Our findings of the electrical conductivity of antigorite obtained prior to dehydration are consistent with those reported by Guo *et al*.^27^ but are higher than those reported by ref.^26^. This inconsistency is mainly due to the compositions of the samples. Post-dehydration chlorite minerals show high electrical conductivities (close to 1 S/m) attributable to the inclusion of 14% interconnected, conductive, impure magnetite rather than aqueous fluid. The electrical conductivity of interconnected aqueous fluids derived from the dehydration of chlorite is less than 0.01 S/m. Thus, although chlorite is a major host of water within a subducting slab, it is impossible to build a causal relation between the dehydration-induced aqueous fluids and the anomalously high observed electrical conductivity values.

**Figure 6:**
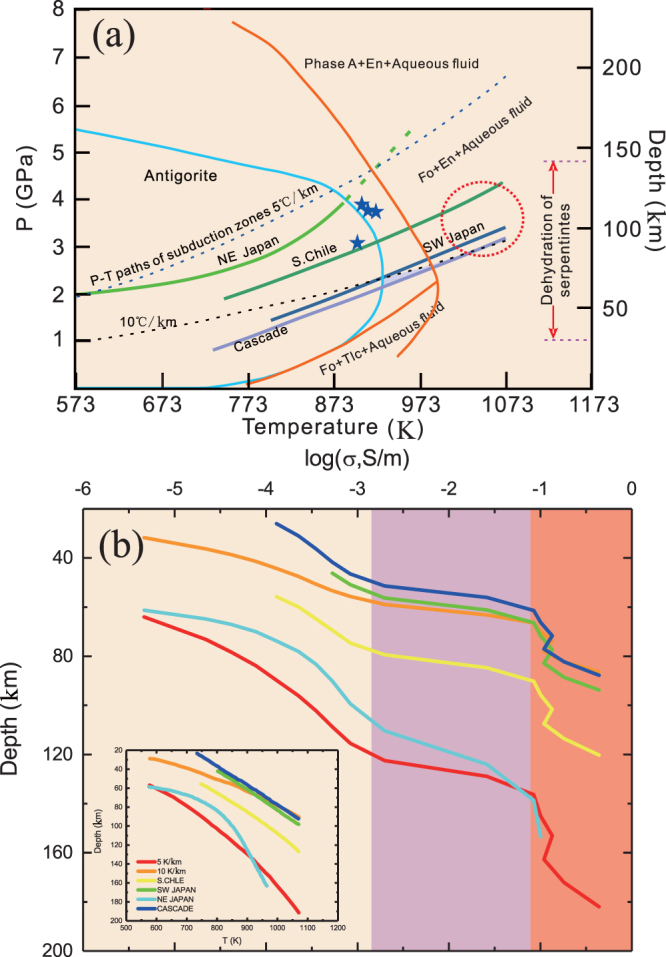
Stability of antigorite, pressure-temperature paths of subduction zones (**a**), and an electrical conductivity model based on laboratory data (**b**). **(a)** Stability of antigorite and pressure-temperature paths of subduction zones. Modified after Deschamps *et al*.^38^. The stability field of antigorite was reproduced from the investigations by Wünder and Schreyer (cyan line) and Ulmer and Trommsdorff (orange line). The solid curves represent different P-T paths of subduction zones (data are sourced from Fukao *et al*.^43^, Furukawa^44^, and Peacock and Wang^1^). Dashed lines are geothermal gradients for subducted oceanic crust having different ages. Stars represent earthquakes. Red-dotted circle is the high-conductivity anomaly region. **(b**) A model of laboratory-based conductivities for predicting the electrical conductivities for slabs in different subduction zones. Conductivity-depth profiles established from the data for antigorite from this study. Thick, solid lines denote the electrical conductivity profiles from the data for antigorite from this study for both hot and cold subduction zones, including South Chile (yellow line), southwestern Japan (green line), Northeast Japan (cyan line) and Cascadia (blue line). The electrical conductivities along different geothermal gradients of 5 K/km (red line) and 10 K/km (orange line) are also shown. The calculated results show high-conductivity zones in the mantle wedge at depths of 60–180 km. The lateritious regions represent anomalous high-conductivity zones detected by MT surveys.

MT studies have demonstrated that conductive anomalies at shallow depths in subduction zones (i.e., at depths of less than 40 km) can be attributed to the accumulation of aqueous fluids (i.e., seawater)^15^ and melt^10^. Regions with relatively strong conductive anomalies in the mantle wedge at depths of ~40–200 km are probably related to the dehydration to the subducting slab. Electrical conductivity models displaying features related to the release of fluids at depth must be consistent with geothermal and petrologic models. The potential for dehydration of a subducting slab is strongly dependent on the geothermal gradient and the stability of key hydrous phases such as antigorite and chlorite (Fig. 6a). Since chlorite can be ruled out as a possible factor, we assumed that the dominant mineral in the subduction at depths of 40–200 km was antigorite. We consequently calculated the conductivities of subducting slabs using several different geothermal gradients. Figure 6b shows a conductivity-depth model based on the presence of antigorite at different geothermal gradients.

In the slabs of cold subduction zones^9^ (e.g., Northeast Japan), the dehydration of antigorite occurs at depths below 150 km^38^. An MT profile revealed high-conductivity anomalies at depths of ~150–200 km (Fig. 1) corresponding to the depth of dehydration of antigorite. Considering our modelled conductivity values along different geothermal gradients in conjunction with the petrologic findings, we predict that the high-conductivity anomalies occur at depths of 150–190 km, which is consistent with the MT results. In contrast, the relatively young subducted slabs (less than ~30 Myr) imaged in relatively hot environments, such as in the southwestern Japan, South Chile and Cascade subduction arcs, are associated with a high geothermal gradient^1,43–45^. When the temperature exceeds ~923 K, which corresponds to depths of ~60 km in those subduction zones, antigorite decomposes into forsterite, enstatite and aqueous fluids^2,22,33^. According to the results of MT surveying^5,7,8,10–15^ within the southwestern Japan, South Chile and Cascade subduction arcs (Fig. 1), high conductivities reaching 1 S/m were observed at depths of 60–150 km (i.e., in the mantle wedge region), which coincides with the depth of dehydration of antigorite (Fig. 1). Our models show that the range of electrical conductivity values of 0.1–1.0 S/m corresponds to depths of 60–130 km, which effectively agrees with the electrical conductivity estimates based on geophysical observations. Therefore, the dehydration of antigorite is the most plausible explanation for the high-conductivity anomalies within the abovementioned regions in the slabs of both cold and warm subduction zones.

In addition, our study suggests that the enhancement of the electrical conductivity of antigorite following the dehydration-induced release of aqueous fluid is much more effective than the introduction of additional high-salinity fluids into a “dry” antigorite system^26^. According to the high geothermal gradient required for the generation of melt, partial melt can be eliminated as a possible influence on high-conductivity anomalies.

In summary, we provide strong evidence for the high conductivities of dehydration-induced aqueous fluids, which can enhance the bulk electrical conductivity of antigorite. Most high-conductivity regions associated with subduction zones could be attributed to such fluids that are released during the dehydration of antigorite and the subsequent formation of an interconnected network of those conductive fluids.

## Methods

### Starting materials

Both natural and synthetic antigorite samples were selected to study their electrical conductivity. The natural sample was a massive antigorite from Nome, Nagasaki, Japan. It contained approximately 5–10% magnetite, which presented as isolated crystals within the antigorite matrix (shown in Fig. 4). The chemical compositions obtained via electron microprobe analysis are shown in Table S1.

The starting synthetic sample was a crystal of antigorite that was selected from the natural massive antigorite and subsequently crushed into fine grains. A powder sample with a grain size of less than 5 μm was packed in a Mo capsule and hot-pressed at 3 GPa and 773 K for 2 hours using a 1000-tonne Walker-type uniaxial split-cylinder apparatus installed in the High Pressure Laboratory at the Mineral Physics Institute, Stony Brook University. Both prior to and following the electrical conductivity measurements, the natural and hot-pressed samples (Fig. 4) were characterized using scanning electron microscopy (SEM) in addition to Fourier-transform infrared (FTIR) and Raman spectroscopy.

### Impedance spectra measurements

High-pressure conductivity experiments were performed in a Walker-type multi-anvil apparatus using a 14/8 assembly consisting of one MgO octahedral pressure medium with a 14-mm edge length and eight tungsten carbide cubes with 8-mm corner-truncation edge lengths. The complex impedance spectroscopy studies were conducted within a frequency range from 1 MHz to 0.1 or 0.01 Hz with a voltage of 1 V using a Solartron 1260 impedance/gain-phase analyser at 3 GPa and 4 GPa at temperatures ranging from 523 K to 1073 K. The samples were cut into cylindrical shapes with diameters of ~1.7 mm and thicknesses of ~0.6–1.0 mm. The samples were sandwiched between Mo electrodes surrounded by alumina rings. The samples were insulated with a boron nitride or MgO sleeve. The oxygen fugacity was controlled by the Mo-MoO_2_ buffer. The temperature was monitored using a W5%Re-W26%Re thermocouple in contact with the Mo electrodes. The electrical conductivities of the antigorite samples were measured during several heating and cooling cycles at high temperature and pressure. Complex impedance data were collected during three heating and cooling cycles from 500 K to 1073 K at 3 and 4 GPa.

The temperature dependence of the electrical conductivity of antigorite during three stages (i.e., prior to dehydration, during dehydration and after dehydration) can be described by an Arrhenius-type relation:

4 $$\sigma =A\,\exp (-\frac{{\rm{\Delta }}H}{{\rm{RT}}})$$

where A is a pre-exponential factor, ΔH is the activation enthalpy, R is the gas constant and T is the temperature.

The electrical conductivities of the natural and hot-pressed antigorite samples were measured at 4 GPa and 3 GPa, respectively, from 523 K to 1073 K. The conductivity values were calculated from the complex impedance spectra of the samples **(**Fig. S2). The first and second cycles were performed within the stability field of antigorite to avoid dehydration, and the electrical conductivity of antigorite was reproducible after the first heating cycle. The third cycle explored the conditions outside of the stability field of antigorite to investigate the effects of dehydration on the electrical conductivity.

## Electronic supplementary material

Supplementary Information
