# Supplementary material for: Constraints from the dehydration of antigorite on high-conductivity anomalies in subduction zones
Source: Sci Rep. 2017 Dec 4;7:16893. doi: 10.1038/s41598-017-16883-4 (PMC5714963; doi:10.1038/s41598-017-16883-4)
Supplement: Supplementary file 1 — Supplementary Information [file 41598_2017_16883_MOESM1_ESM.pdf]

Supplementary materials for "Constraints from the dehydration of antigorite on high-conductivity anomalies in subduction zones"

Duojun Wang[1,2*], Xiaowei Liu[3†], Tao Liu[1†], Kewei Shen[1], David O. Welch[4], Baosheng Li[2]

1.College of Earth Sciences, University of Chinese Academy of Sciences; Beijing, 100049, China

2. Mineral physics institute, State university of New York at Stony Brook, Stony brook, 11790, USA

3. Laboratory of Mechanics on Disaster and Environment in Western China, Lanzhou University, Lanzhou, 730000, China

4. Department of Condensed matter physics and Materials Science, Brookhaven National Laboratory, Upton, 11793, USA

*Corresponding author Email:duojunwang@ucas.ac.cn

†these authors contributed equally to this work

Figure S1 FTIR spectra of natural (a) and hot-pressed antigorite before and after electrical conductivity measurements

The red and green lines show the results before and after the electrical conductivity measurements were collected, respectively.

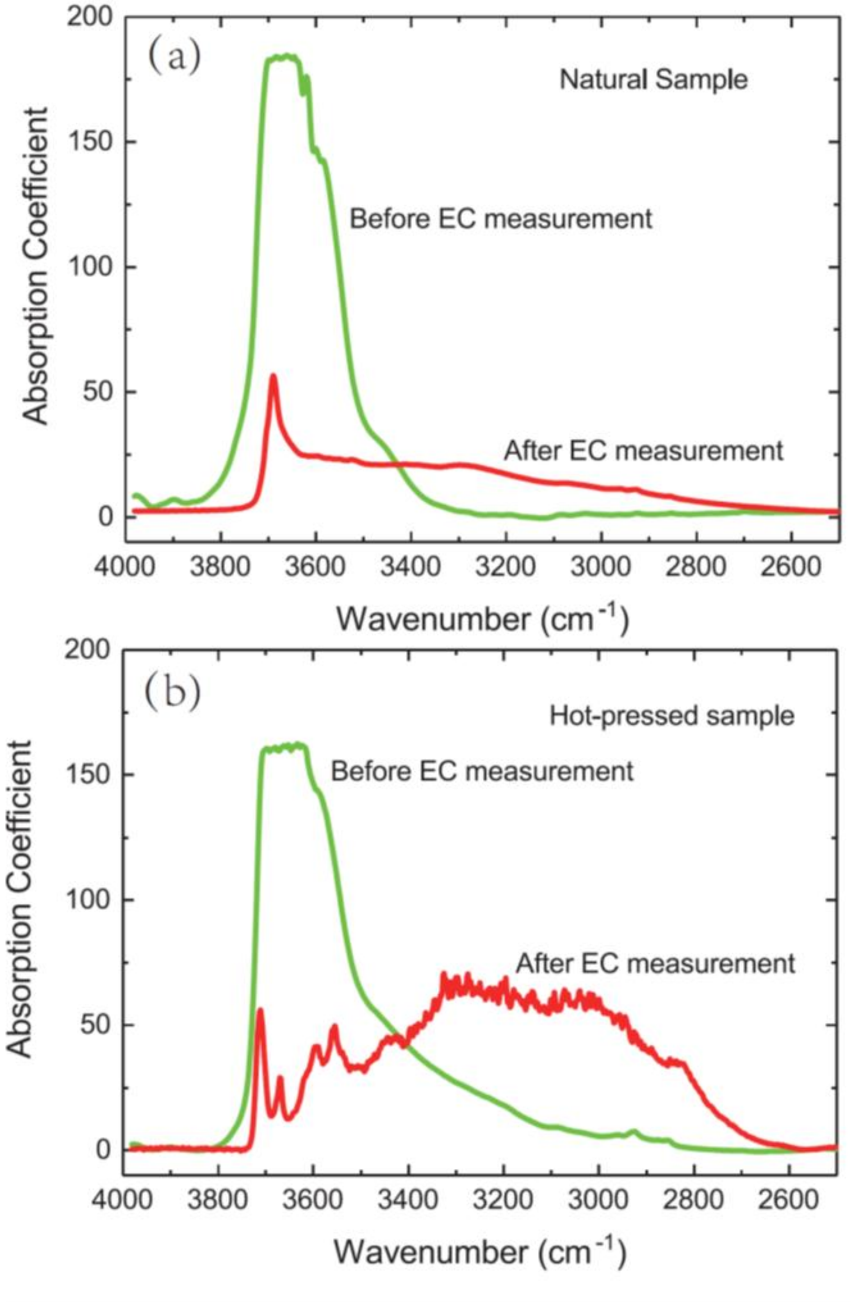

Figure S2 Impedance spectra prior to dehydration (a)、during dehydration (b)\after dehydration(c) for antigorite at 4 GPa.

Z' and Z'' are the real and imaginary values.

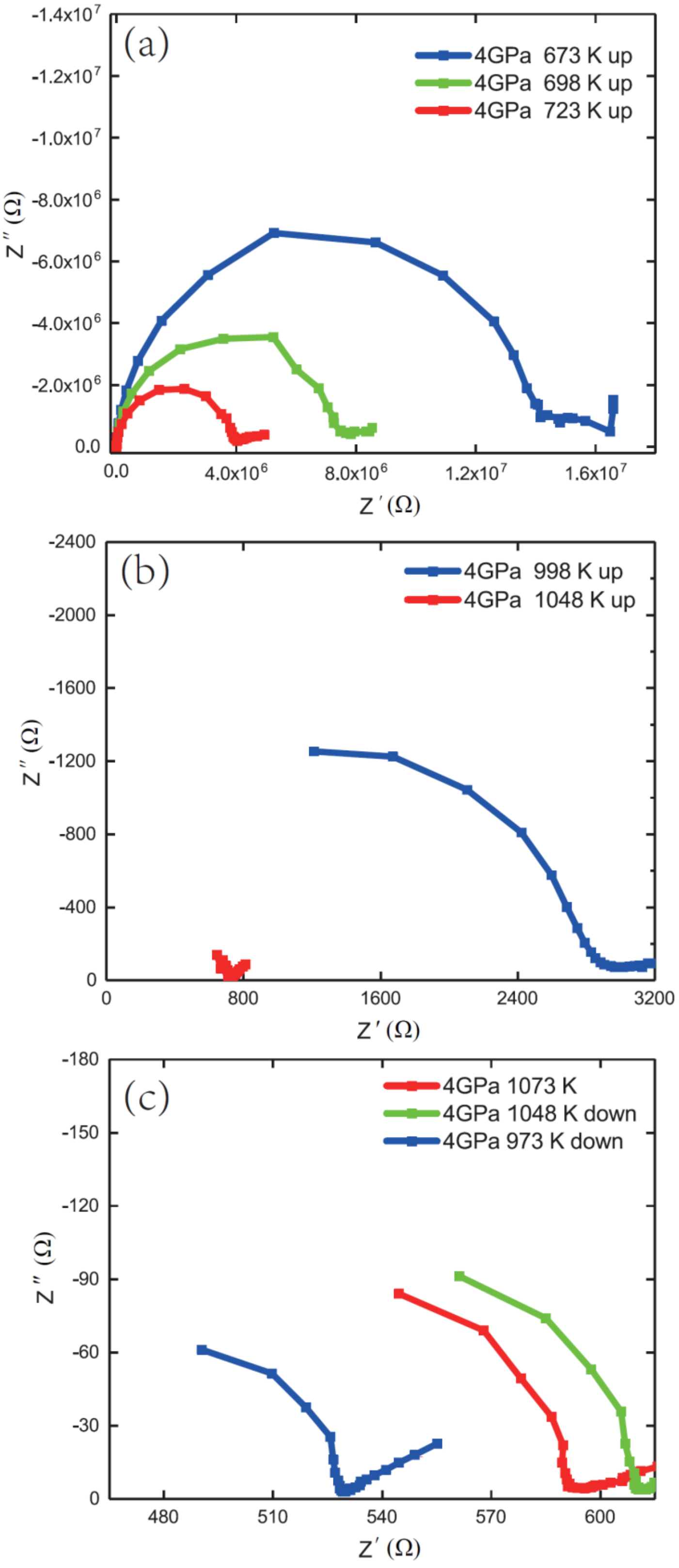

Table S1

The chemical composition of antigorite determined via electron microbe analysis

Antigorite	Wt (%)
MgO	39.14
Al_2O_3	1.03
CaO	0.02
TiO_2	0.01
SiO_2	43.04
NiO	0.06
MnO	0.06
FeO	2.39
Total	85.75
